# Increased arterial pressure volume index and cardiovascular risk score in China

**DOI:** 10.1186/s12872-022-03035-4

**Published:** 2023-01-16

**Authors:** Lin Jin, Mengjiao Zhang, Lei Sha, Mengmeng Cao, Lanyue Tong, Qingqing Chen, Cuiqin Shen, Lianfang Du, Liping Liu, Zhaojun Li

**Affiliations:** 1 Department of Ultrasound, Guanghua Hospital Affiliated to Shanghai University of Traditional Chinese Medicine, Shanghai, 200052 China; 2 Department of Ultrasound, First Hospital of Shanxi Medical University, Taiyuan, 030001 Shanxi China; 3 Department of Medical Imaging, Weifang Medical University, Weifang, 261053 Shandong China; 4 Department of Ultrasound, Shanghai General Hospital Jiading Branch, Shanghai Jiaotong University School of Medicine, Shanghai, 201803 China; 5 Department of Ultrasound, Shanghai General Hospital, Shanghai Jiaotong University School of Medicine, 100 Haining Road, Hongkou District, Shanghai, 200080 China

**Keywords:** Arterial stiffness, Cardiovascular disease, Arterial pressure volume index, Risk assessment

## Abstract

**Background and objective:**

The new non-invasive arterial stiffness indices, arterial pressure volume index (API) is explored as a novel marker of residual stress in the wall of the peripheral muscular arteries at zero-stress state in clinical settings. The present study aimed to study the association of API with cardiovascular disease (CVD) risk in China (China-PAR).

**Methods:**

According to China-PAR score, participants were divided into three groups: low risk (< 5%), medium risk (5–9.9%), and high risk (≥ 10.0%). API ≥ 31 was defined as high API, and the incidences of high API were compared. Logistic regression models were used to analyze the risk factors of high API and high risk China-PAR categories. The association between China-PAR and API was analyzed by restrictive cubic spline.

**Results:**

The study included 4311 participants. After adjustments for confounding factors, high API was independent factor associated with high risk China-PAR categories, and the probability of high API was 1.366 times higher than that in normal API subjects. While, the independent factors associated with high API were BMI, blood pressure and heart rate. Furthermore, API had a significant U-shaped association with China-PAR. CVD risk was lowest with API of 19 units, the fastest increase at 26 units and the flattest starting point at 59 units.

**Conclusion:**

API, an indicator of arterial stiffness and residual stress, had a U-shaped association with China-PAR score and might play an important role in predicting CVD risk in Chinese natural populations.

## Background

Several studies reveal that measures of vascular function damage are powerful predictors of cardiovascular disease (CVD) risk [1]. Arterial stiffening is central in the vascular aging process, and increased arterial stiffness is an independent predictor of CVD events [2, 3]. In addition, a number of studies have shown high arterial stiffness was associated with multiple organ damages, such as Systemic Hemodynamic Atherosclerotic Syndrome [4] and cognitive function decline [5].

Novel markers of arterial stiffness, such as arterial velocity pulse index (AVI) and arterial pressure volume index (API), are predictive of CVD incidence and progression [6, 7]. AVI and API were measured using cuff oscillometry to evaluate pulse waveforms. AVI reflects stiffness of the central arteries, and an increase in AVI indicates increased arterial stiffness from the aorta to the brachial artery and increased resistance in the peripheral arteries [8]. On the contrary, API reflects the residual stress in the peripheral muscular arteries wall at zero-stress state [9]. Residual stress is a key factor in maintaining the normal physiological function and physiological state of arterial tissue. Thus, API is closely related to cardiac function, and is significantly and independently associated with CVD risk scores [3, 10, 11]. However, traditionally, vascular research has focused on atherosclerotic vascular disease, whereas arterial stiffness has not attracted similar attention.

CVD risk assessment is a fundamental component of CVD prevention [12]. Several prediction models for CVD risk evaluation have been built and applied in public health and clinical practice in different populations. The Framingham heart study developed first coronary heart disease risk prediction models since 1976 [13], and Framingham cardiovascular disease risk score (FCVRS) is widely used in the world. However, previous studies showed that these equations are likely to overestimate the risk of Chinese population [14, 15], mainly due to the incidence rate and risk factors exposure level of Chinese people are lower than that of Western populations. The prediction for Atherosclerotic Cardiovascular Disease Risk in China (China-PAR) project has recently been developed based on data from multiple contemporary Chinese adult cohorts in 2016 and was widely used in practice [12].

However, at present, little is known about the relationship of vascular function damage and CVD risk scores in large sample natural populations. Therefore, the purpose of the present study was to evaluate the association between API and CVD risk scores by adjusting for potential confounders using the restrictive cubic spline (RCS) functions in Chinese population. Our study also aimed to determine the specific influence of age, gender, anthropometric parameters, and blood pressure (BP) levels on high API.

## Methods

### Study design and population

This cross-sectional study enrolled subjects who underwent health checks at Shanghai General Hospital Jiading Branch, Shanghai, China from August 2020 to December 2020. A total of 4311 subjects (2091males and 2220 females) aged 20–79 years were enrolled.

### Inclusion and exclusion criteria

Individuals who were aged 18 years and above, and who had a good cognitive function and voluntarily signed the informed consent were included. The exclusion criteria included subjects with severe mental illness or pregnancy; subjects with a history of CVD; subjects with upper limb infection, or subjects unable to obtained AVI and API due to previous vascular intervention or limb amputation; subjects who were undergoing hemodialysis or with atrial fibrillation.

### Baseline data collection

A standardized questionnaire was administered by a trained interviewer at baseline to collect information comprising age, sex, personal medical history and history of hypertension, smoking condition and alcohol consumption. Weight and height were obtained according to a standardized protocol, and body mass index (BMI) calculated as weight (kg)/height (m)^2^.

Blood samples were obtained from the subjects after an overnight fast at time of survey, serum separated and stored at − 70 °C. Blood indicators were measured by immunoturbidimetry with automatic biochemical instrument at the time of the survey, including total cholesterol (TC), high density lipoprotein cholesterol (HDL-C), low-density lipoprotein cholesterol (LDL-C), fasting plasma glucose (FPG), triglyceride (TG) levels, etc.

### Arterial stiffness indices

AVI and API were measured using cuff oscillometry with PASESA AVE-2000Pro (Shisei Datum, Tokyo, Japan) by trained technicians. Systolic blood pressure (SBP), diastolic blood pressure (DBP) and heart rate (HR) were also obtained simultaneously. The subjects shall rest for at least 5 min and empty the bladder before measurement, stop smoking and coffee at least 24 h before the examination. The subjects were in the sitting position, and measurements were taken in a quiet, temperature controlled room (24–26 °C). Then a cuff was wrapped around one-side of the upper arm. The balloon mark was aligned with the brachial artery, and the lower edge of the cuff was 2  cm away from the transverse line of the cubital fossa.

As Hitsumoto [16] and Sasaki-Nakashima [11] described previously, AVI was calculated as 20 × (Vr/Vf). Vf was measured as the first peak of the differentiated waveform between pulse wave and time, while Vr was the absolute value of the bottom of the differentiated waveform between pulse wave and time. API was defined as the time series of occluded cuff pressures and the amplitudes of pulse oscillations measured according to the slopes of the local curve between the cuff pressure reduction and the corresponding arterial pressure volume [9, 17]. AVI and API were dimensionless indicators. Rest for 2 min and measure again. Take the average value of three times as the final result.

### Cardiovascular disease risk score

The 10-year risk of CVD was estimated for each individual using the China-PAR algorithms through the evaluation tool research on the website (https://www.cvdrisk.com.cn). China-PAR risk assessment models included in: gender, age, current residence (city or rural), geographical area (North or South, Yangtze River bound), waist circumference, TC, HDL-C, BP, the history of hypertension, diabetes, smoking and the family history of cardiovascular disease. According to predicted CVD 10-year risk, participants were divided into three categories: low risk is defined as < 5.0%, medium risk is defined as 5.0–9.9%, and high risk is defined as ≥ 10.0%.

### Statistical analysis

The continuous variables were presented as mean ± standard deviation (SD), and categorical variables were presented as numbers or percentages, or as median and interquartile range if the distribution did not appear to follow a normal distribution. SPSS 22.0 (IBM, Armonk, NY, USA) statistical analysis software was used. The continuous data were compared using variance analysis for inter group comparison. The categorical variables between groups were compared by chi square test.

High API status was defined as API ≥ 31 [18]. Taking high API and high risk China-PAR categories as variables of two classification levels, a stepwise multivariate logistic regression model was used to analyze the risk factors of high API and high risk China-PAR categories respectively.

RCS analyses were used to detect the possible non-linear dependency of the relationship between China-PAR and AVI or API value, using 5 knots at prespecified locations according to the percentiles of the distribution of AVI or API, the 5th, 25th, 50th, 75th, and 95th percentiles [19]. The RCS analyses were carried out using Stata 12.0 (StataCorp, College Station, TX). *p* < 0.05 represented that the difference was statistically significant.

## Results

### Baseline characteristics

The baseline characteristics of the 4311 subjects were shown in Table 1. The average age of subjects was 57.8 ± 12.8 years, and 49% of the participants were male. The mean (± SD) API in subjects was 29.36 ± 7.21 units, and the median was 28 units. In this study, there were 1660 subjects of high API and 2651 subjects of non-high API.

**Table 1:** Basic characteristics of 4311 subjects

Item	Low-risk group (n = 2270)	Medium-risk group (n = 659)	High-risk group (n = 1382)	*p *value
Male, n (%)	497 (21.9%)	325 (49.3%)	1269 (91.8%)	< 0.001
Age (years)	50.60 ± 12.31	62.36 ± 8.32*	67.44 ± 6.21*#	< 0.001
Current smoker, n (%)	67 (3.0%)	35 (5.3%)	156 (11.3%)	< 0.001
Alcohol consumption, n (%)	31 (1.4%)	15 (2.3%)	75 (5.4%)	< 0.001
Hypertension, n (%)	938 (41.3%)	449 (68.1%)	1020 (73.8%)	< 0.001
Diabetes mellitus, n (%)	383 (16.9%)	184 (27.9%)	471 (34.1%)	< 0.001
Dyslipidemia, n (%)	591 (26.0%)	226 (34.3%)	424 (30.7%)	< 0.001
*Medications, n (%)*				
Antihypertension, n (%)	522 (23.0%)	275 (41.7%)	666 (48.2%)	< 0.001
Antidiabetes, n (%)	268 (11.8%)	131 (19.9%)	332 (24.0%)	< 0.001
Height (cm)	162.02 ± 7.64	163.91 ± 8.18*	168.15 ± 6.98*#	< 0.001
Weight (Kg)	64.05 ± 12.84	66.30 ± 11.43*	68.90 ± 10.51*#	< 0.001
Body mass index (kg/m^2^)	24.29 ± 3.83	24.60 ± 3.35	24.34 ± 3.24	0.134
Systolic blood pressure (mm Hg)	125.19 ± 19.98	138.34 ± 22.91*	142.61 ± 24.02*#	< 0.001
Diastolic blood pressure (mm Hg)	78.20 ± 12.56	81.98 ± 13.94*	82.06 ± 13.85*	< 0.001
Heart rate (beats/min)	80.17 ± 12.54	79.19 ± 12.37	79.27 ± 13.00	0.057
*Basic parameter*				
Total cholesterol (mmol/L)	4.55 ± 0.97	4.52 ± 1.10	4.31 ± 1.08*#	< 0.001
Triglyceride (mmol/L)	1.43 ± 0.98	1.61 ± 1.14*	1.56 ± 1.01*	< 0.001
HDL cholesterol (mmol/L)	1.19 ± 0.33	1.10 ± 0.29*	1.00 ± 0.26*#	< 0.001
LDL cholesterol (mmol/L)	2.85 ± 0.90	2.85 ± 1.03	2.75 ± 1.02*	0.009
Fasting plasma glucose (mmol/L)	5.59 ± 1.56	5.96 ± 1.72*	6.23 ± 2.05*#	< 0.001
AVI	16.40 ± 5.98	19.05 ± 6.14*	19.84 ± 6.50*#	< 0.001
API	27.37 ± 6.15	30.56 ± 7.51*	32.05 ± 7.66*#	< 0.001
China-PAR score	0.01(0.00, 0.02)	0.06(0.05, 0.08)*	0.16 (0.12, 0.20)*#	< 0.001

Compared with the low-risk group, *p <  0.05; Compared with the medium-risk group, #*p * < 0.05

### Partial correlation analysis among age, SBP, HR, China-PAR score and API adjusted by risk categories

**Fig. 1:**
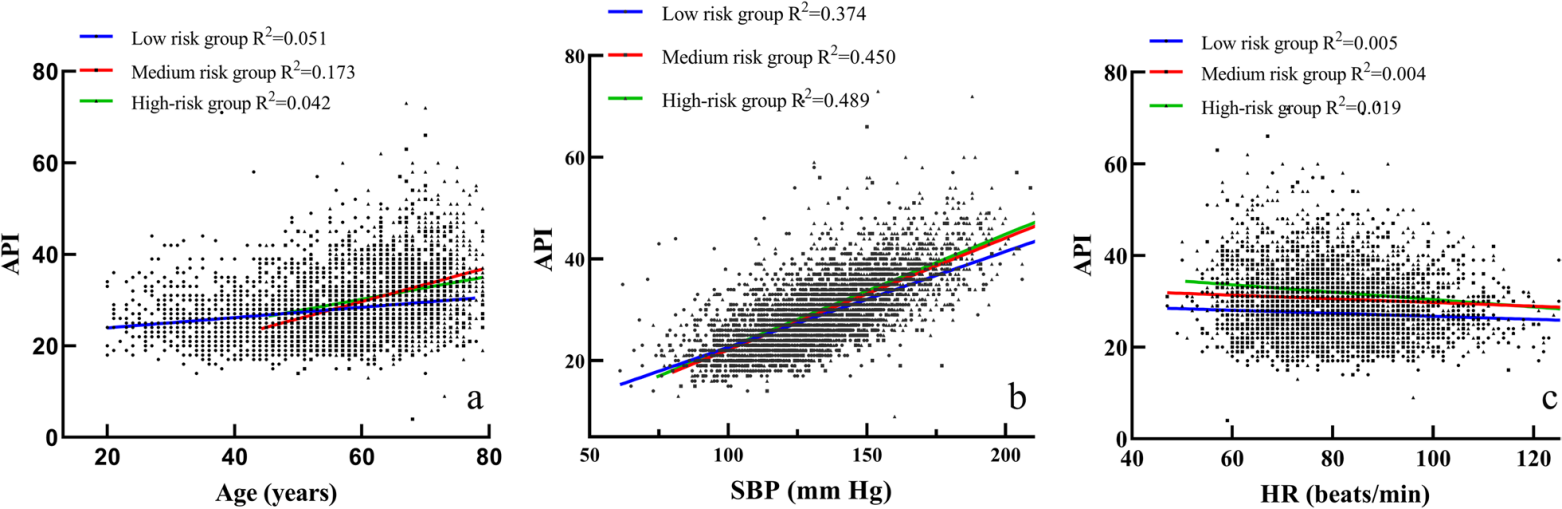
Scatter plot and linear regression curve of API with age, systolic blood pressure and heart rate. **a** API was positively correlated with age in each group; **b** API was positively correlated with systolic blood pressure in each group; **c** API was negative correlated with heart rate in each group

API, a marker of muscular arteries residual stress, was significantly higher in medium and high-risk participants than in low-risk participants. In the linear regression analysis, API was positively correlated with age and SBP in each group, and negative correlated with HR (Fig. 1). Furthermore, API was significantly and positively associated with China-PAR score in low-risk group (*r* = 0.307, *p* < 0.001) and high-risk group (*r* = 0.237, *p* < 0.001). No significant difference was found between API and China-PAR score in medium risk group (*p* = 0.535).

### Independent factors associated with high API

After adjustments for confounding factors, multivariate logistic regression model analyzed the risk factors of high API in this study. BMI, SBP, DBP, HR, hypertension and antihypertension medication were independent risk factors for high API (Table 2).

**Table 2:** Stepwise multivariate regression analysis for high API

	β	S.E	Wald	*p* value	OR	OR 95% CI	
						Lower limit	Upper limit
BMI	0.057	0.014	17.568	< 0.001	1.058	1.031	1.087
SBP	0.152	0.005	880.860	< 0.001	1.165	1.153	1.176
DBP	− 0.157	0.006	625.897	< 0.001	0.854	0.844	0.865
Hypertension	0.763	0.155	24.236	< 0.001	2.145	1.583	2.907
Antihypertension medication	− 0.309	0.132	5.471	0.019	0.734	0.567	0.951
Heart rate	− 0.014	0.004	13.142	< 0.001	0.986	0.978	0.994

Confounders: (baseline) age, BMI, SBP, DBP, heart rate, fasting plasma glucose, history of smoking, alcohol consumption, hypertension, diabetes mellitus and medications of antihypertension. β is the regression coefficient; S.E. is the standard error; Wald is the Chi-square value; OR is the odds ratio

### Incidence of high API

The comparisons of the incidence of high API in different age and gender subgroups were showed in Table 3. There were significant differences in the incidence of high API in both 18–44 years old and 45–59 years old subgroups (*p* ≤ 0.05). And the incidence of high API was higher in women than in men in low and medium risk groups, and lower in men in the high-risk group.

**Table 3:** Comparison of the incidence of high API in different age and gender

	Low-risk group (n = 2270)	Medium-risk group (n = 659)	High-risk group (n = 1382)	*p* value
Total, n (%)	628 (27. 7%)	309 (46.9%)	723 (52.3%)	< 0.001
*Age, n (%)*				
18–44	135 (6.0%)	0 (0.00%)	0 (0.0%)	< 0.001
45–59	232 (10.2%)	57 (8.7%)	61 (4.4%)	< 0.001
≥ 60	261 (11.5%)	252 (38.2%)	662 (47.9%)	0.098
*Gender, n (%)*				
Male	106 (21.3%)	57 (17.5%)	619 (48.8%)	< 0.001
Female	522 (29.4%)	252 (75.5%)	104 (92.0%)	< 0.001

### Correlations of API and AVI with China-PAR score

In stepwise multivariate regression analysis including male, BMI, SBP, DBP, HR and high API, the odds ratios for high risk China-PAR increased across high API, reaching 1.366 (95% confidence interval [CI], 1.060–1.759) (Table 4).

**Table 4:** Logistic regression analysis of high risk China-PAR categories (stepwise)

	β	S.E	Wald	*p* value	OR	OR 95% CI	
						Lower limit	Upper limit
Male	4.293	0.139	952.532	< 0.001	73.209	55.738	96.155
BMI	− 0.085	0.014	37.140	< 0.001	0.918	0.893	0.944
SBP	0.060	0.004	267.253	< 0.001	1.062	1.054	1.069
DBP	− 0.064	0.005	159.911	< 0.001	0.938	0.928	0.947
High API	0.312	0.129	5.806	0.016	1.366	1.060	1.759

Confounders: (baseline) male, BMI, SBP, DBP, Heart rate and high API. β is the regression coefficient; S.E. is the standard error; Wald is the Chi-square value; OR is the odds ratio

**Fig. 2:**
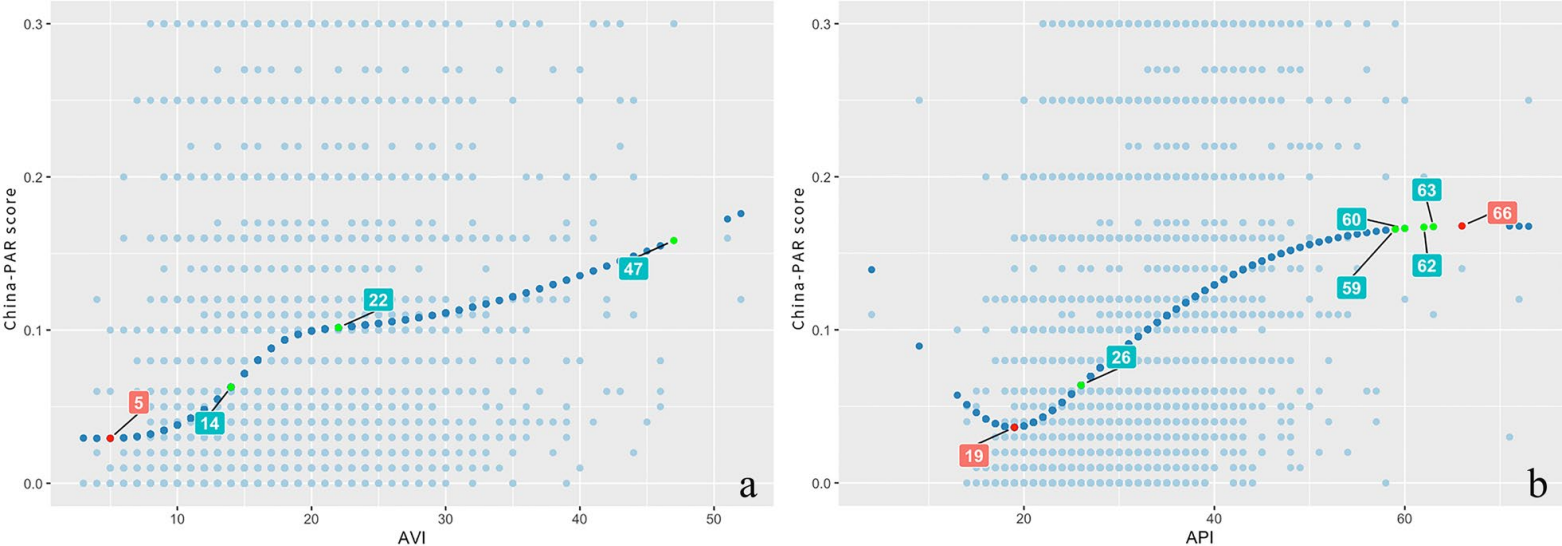
Relationships between AVI, API and China-PAR score based on Restricted Cubic Spline Functions. **a** AVI showed a significant U-shaped relationship with the China-PAR score, with AVI values of 5 units associated with the lowest CVD risk score. **b** There was a significant U-shaped relationship between API and China-PAR scores, with the API value associated with the lowest CVD risk score was 19 units

In order to further explore the relationships between API, AVI and China-PAR score, RCS analyses were used and the curves of API, AVI and China-PAR score were drawn. The results showed that: Both AVI and API had significant U-shaped associations with China-PAR score. For AVI, the China-PAR score increased from 5units and the increase in China-PAR score was steeper after 14 units. When AVI reached 22 units, the increase in China-PAR score showed a relatively flat trend (Fig. 2a). In parallel, for API, China-PAR score started to increase from 19 units and after 26 units the increase in China-PAR score was steeper. When API reached 59 units, the increase in China-PAR score showed a relatively flat trend (Fig. 2b).

## Discussion

The study was to evaluate the relationship between API and CVD risk score as defined by China-PAR score. Stepwise multivariate regression analysis showed that API was independent risk factor for high risk China-PAR categories. Furthermore, we observed a significant U-shaped association between API and China-PAR score, the lowest risk score for CVD was when API was at 19 units, after 26 units, the increase in China-PAR score was steeper, and when API reached 59 units, the increase in China-PAR score showed a relatively flat trend.

The typical pathological change of arteriosclerosis is vascular structure and function change, which is the pathologic base of the cardiovascular and cerebrovascular disease such as myocardial infarction and brain stroke [20, 21]. Therefore, the identification of vascular alterations at the subclinical stage can potentially facilitate the screening, prevention, and risk stratification of CVD [22]. Previous studies have demonstrated that the residual stress can prevent the stress concentrations and maintain vessel compliance, so it is necessary to understand the stress-growth relationship in the zero-stress state of the vessel wall [8]. API is a novel indicator of the residual stress at zero-stress state, reflects reactive vasodilation and stiffness of peripheral muscular arteries [9, 23], and provides a different perspective for assessing of mechanical vascular wall properties of the arterial tree. In the present study, high CVD risk categories was associated with high API independent of other significant factors. In addition, it should be noted that BMI, HR, BP (systolic, diastolic) and antihypertension medication were independent factors of high API.

Furthermore, our study pointed out a significant U-shaped relationship between API and China-PAR risk score. There were several possible mechanisms by which increased API induced nonlinear increased in China-PAR risk score. First, API increased with age [24], while the relationship between age and arterial stiffness was more appropriately expressed by a nonlinear model than by the traditional linear model approach [20]. A possible explanation was that the arterial walls presented viscoelastic biomechanical properties and exhibited the nonlinear stress-stain relations. With age, the ratio of collagen/elastin in the arterial wall increased. When adverse remodeling of arterial wall structure and function occurred, it can induce arterial stiffness and change the residual stress of the arterial wall [25]. As a result of residual stress, the arterial wall was not uniformly compressed, with the inner layer in compression and the outer layer in tension, making the middle layer the most compressed [26]. Second, blood pressure (BP) can affect both arterial stiffness and CVD risk. Studies have shown that BP and CVD risk have a J-curve phenomenon, which means that the risk of CVD may increase both when BP is too high and too low [27]. Similarly, there was the nonlinear relationship between BP and arterial wall stress [28]. With the increase of BP, the pressure load on the tube wall increased, resulting in the rupture of elastic fibers and the increase of collagen fibers, which changed the residual stress and reduced the compliance of the artery [29].

## Limitations

This study has several limitations. First, this study was a single center and cross-sectional study without end-point follow-up. However, we had a larger sample size, and in the restrictive cubic spline model, the predictive value of AVI and API remained significantly associated with an increased risk of China-PAR in Chinese natural populations. Second, at present, API is not the gold-standard method for evaluating arterial stiffness. However, it has been reported that API reflects the residual stress in the wall of the peripheral muscular arteries at zero transmural pressure and is correlated with other established markers of arterial stiffness [7]. Therefore, multi-center cohort study and objective index studies of vascular structure are needed to validate the preliminary results in the future.

## Conclusion

In conclusion, as an indicator of arterial stiffness, API may be used as a predictor of CVD risk to provide important information for clinical practice. Our results showed that API had a significant U-shaped association with China-PAR score, with the lowest CVD risk score was 19 units, and the fastest increasing CVD risk occurred after API was 26 units. Non-invasive arterial stiffness indices would help to identify individuals with different CVD risk categories who may benefit from more aggressive management.

## Data Availability

All data generated or used during the study appear in the submitted article.
